# Nose and Throat Affections

**Published:** 1900-03

**Authors:** C. H. Powell

**Affiliations:** Professor of Physical Diagnosis and Clinical Medicine Barnes Medical College, St. Louis, Mo.


### NOSE AND THROAT AFFECTIONS.

BY C. H. POWELL, A.M., M.D.,

Professor of Physical Diagnosis and Clinical Medicine Barnes Medical College, St. Louis, Mo.

Now that the season of the year is upon us at which time disturbances of the nose, throat and lungs are predominating, an article calling attention to the different affections, their causes, pathological characteristics, and the treatment, is certainly of practical importance. Nasal involvement is certainly the most common, morbid phenomenon we meet with of every degree from simple congestion of the Schneiderian membrane to ulceration, hypertrophy, and deflection of the nasal septum. One of the first indications that a trouble exists in the nose is indicated by the presence of nasal discharge. The most common discharge observed occurs from a simple cold, or more technically termed, an acute rhinitis. This troublesome affection usually begins as a dryness of the Schneiderian membrane which has as an accompanying condition a vaso-motor constriction, the blood-vessels are accordingly emptied of their contents, and as a consequence the function of the parts supplied by the blood is temporarily suspended. This cessation of function, however, does not persist longer than twelve or twenty-four hours, when the blood-vessels resume their function, but to an extent greatly in excess of what previously existed; there is then a greater amount of blood carried to the parts, and as a result we have the escape of the watery element manifesting itself in the shape of a large discharge of mucus, which, from the fact that it follows an inflammatory process, is, in a strict sense, an exudation—not a transudation. This flow of mucus is usually very profuse, causing the patient to be continually using his kerchief which is saturated with the discharge. The attack is often ushered in by repeated attacks of sneezing, headache and a sensation of fulness in the frontal sinus. These unpleasant sensations usually are markedly relieved as soon as the discharge of mucus is well established. This first state of morbid activity on the part of the nose gives us in a most typical form the symptoms described as an acute nasal catarrh. For the relief of such an acute condition the indications to be met consist in applying an agent or agents that will constrict the blood-vessels, and at the same time destroy the germs associated with the discharge. Having accompanied this, the inflamed mucus should be protected from the atmospheric irritants. As the treatment usually employed by me is quickly, and, often, permanently satisfactory in correcting this trouble, I will outline the *modus operandi.* In the first place I provide myself with a Mulhall-Warner nasal douche, which has a long curved tip for insertion up behind the uvula. I find this simple instrument of signal utility in the treatment of all nasal difficulties. I now prepare a solution one part of Glyco-Thymoline (Kress) to six parts of lukewarm water, and wash out the nose thoroughly; having accomplished this I next spray the nares thoroughly with a solution of cocain, ten grains to the ounce, and then complete the treatment by throwing into the nose with a DeVilbiss instrument some heated vaseline, which soothes, protects and keeps the constricting influence of the cocain solution *in situ* for a considerable interval. I seldom find it necessary to repeat this treatment more than once or twice. The relief experienced in every instance is prompt, and grateful to the patient. There is another class of patients who are sufferers from a chronic nasal discharge whose condition is due to the occupation engaged in; thus, those who work in factories where the air is continually loaded with dust and débris, being constantly exposed to the irritating influence of these particles, the nasal disturbance is perpetuated. In this class of cases the same plan of action is preferable, namely, first thorough cleansing of the nose with Glyco-Thymoline solution in the same strength as before and the after application of an unguent. A very useful remedy that gives well-marked beneficial results is a four per cent. menthol and alboline mixture, which is a very efficient and cooling ointment that, being liquid, can be readily sprayed upon the nasal mucous membrane. There is still another class of cases that are very persistent in resisting different kinds of treatment. I refer to what is technically known among the people as chronic nasal catarrh. In this class of subjects the pathological state of the nose is to be carefully inquired into, as the catarrhal discharge is only a symptom of something else; this something else constitutes the disease to be treated. In one case it will be found to be an hypertrophic rhinitis, in another a stenosis of one of the nares from a deflected septum, and in a third case, especially in the case of a child, the presence of a foreign body, a tumor or a polypus. Treatment of the nasal discharge, therefore, to be efficacious, should consist in directing attention to whichever one of these or other conditions are found to be responsible for the catarrh. In all cases, however, due to whatever cause the action of Glyco-Thymoline (Kress) has impressed me most favorably. I will outline a half-dozen cases wherein I have derived the most gratifying results from this remedy :

CASE I.—*Acute Rhinitis.*—A lady consulted me with a very bad cold, which had been persisting, to her intense annoyance, for several days. She called upon me in the belief that the difficulty was increasing instead of diminishing. Posterior nasal washing was at once done, using Glyco-Thymoline (Kress) in warm water, one part to six, and the nasal fossæ sprayed with cocain solution. Patient was also given a six-ounce bottle of Glyco-Thymoline (Kress) solution, and advised to apply with an atomizer three times a day. Recovery was prompt and thorough under its use.

CASE II.—*Hypertrophic Rhinitis.*—This case was in a man who worked out doors in all kinds of weather. In addition, he was an inveterate tobacco-user, smoking as many as two dozen pipes of tobacco daily. He was also subject to great relaxation of the pharyngeal structures, had a cough, enlarged tonsils, and was a terrible snorer, sleeping with his mouth open. This patient was treated with Glyco-Thymoline (Kress) in solution, and the throat frequently gargled with a fifty per cent. solution of the same remedy. Internally quinine, iron and strychnia were given him, and he was advised to lessen the amount of tobacco he was consuming. He was also told to dress more warmly. The outcome of the case was very slow, but an uneventful recovery took place.

CASE III.—*Tobacco Pharynx.*—This patient came to me for an annoying cough, which, upon inspection, I discovered was due to an intense hyperemia of the pharyngeal structures. He was given a twenty-five per cent. solution of Glyco-Thymoline (Kress), and instructed to gargle his throat often with the medicine. I saw him a week later, and he advised me that, very much to his own and his wife's delight, the cough had entirely disappeared.

CASE IV.—*Diphtheria.*—I have used Glyco-Thymoline (Kress) in cases of diphtheria with good results. My favorite combination is composed of the following: Glyco-Thymoline 50 per cent., peroxide of hydrogen 50 per cent. For use in the atomizer every hour or so. Although mentioning this under case fourth, I will not specify any particular case, as I employ this prescription in all cases of diphtheria. Of course Glyco-Thymoline (Kress) has no specific influence in diphtheria other than a strong, reliable and harmless antiseptic.

CASE V.—*Chronic Gastritis.*—Having satisfied myself of the signal utility of Glyco-Thymoline in the previous conditions outlined, determined me to test its efficacy in the operation of lavage. A few days ago a conductor on a street railway in St. Louis, came to my office complaining with chronic gastric disease. He had a foul, coated tongue, and said he always felt bloated after meals. I employed lavage, using sodium bicarbonate and Glyco-Thymoline (Kress), two tablepoonsfuls of soda and six tablepoonsfuls of Glyco-Thymoline (Kress) to a quart of hot water. The patient experienced so much relief from this remedy that he returned unsolicited four days later and asked that I repeat the process, which I did. He sent me several other railroad men, and the results in each case were the same.

CASE VI.—*Acute Cystitis.*—A gentleman who sustained an injury to his spinal column from suddenly alighting from his bicycle, had as a result paralysis of his lower extremities, in which his bladder participated—was troubled with acute cystitis and its concomitant evils. For the correction of this his bladder was irrigated with a tablespoonful of Glyco-Thymoline and a tablespoonful of boracic acid to a pint of warm water. It was not necessary to repeat this process more than three times when relief was established, frequent micturition disappeared, and necessity for the continued use of the catheter was obviated.

These cases demonstrate beyond peradventure of a doubt the therapeutic efficacy of Glyco-Thymoline (Kress) as a remedial agent.

